# *In Vivo* Rate of Formaldehyde Condensation with Tetrahydrofolate

**DOI:** 10.3390/metabo10020065

**Published:** 2020-02-12

**Authors:** Hai He, Elad Noor, Perla A. Ramos-Parra, Liliana E. García-Valencia, Jenelle A. Patterson, Rocío I. Díaz de la Garza, Andrew D. Hanson, Arren Bar-Even

**Affiliations:** 1 Max Planck Institute of Molecular Plant Physiology, Am Mühlenberg 1, 14476 Potsdam-Golm, Germany; he@mpimp-golm.mpg.de; 2 Institute of Molecular Systems Biology, ETH Zürich, Otto-Stern-Weg 3, 8093 Zürich, Switzerland; noor@imsb.biol.ethz.ch; 3 Tecnologico de Monterrey, Escuela de Ingeniería y Ciencias, Ave. Eugenio Garza Sada 2501, Monterrey N.L. 64849, Mexico; perlaramos@tec.mx (P.A.R.-P.); liligarciav@tec.mx (L.E.G.-V.); rociodiaz@tec.mx (R.I.D.d.l.G.); 4 Tecnologico de Monterrey, Centro de Biotecnología-FEMSA, Ave. Eugenio Garza Sada 2501, Monterrey N.L. 64849, Mexico; 5 Horticultural Sciences Department, University of Florida, Gainesville, FL 32611, USA; patterson.j@ufl.edu (J.A.P.); adha@ufl.edu (A.D.H.)

**Keywords:** one-carbon metabolism, spontaneous reaction, auxotrophy, serine cycle, phenotypic phase plane

## Abstract

Formaldehyde is a highly reactive compound that participates in multiple spontaneous reactions, but these are mostly deleterious and damage cellular components. In contrast, the spontaneous condensation of formaldehyde with tetrahydrofolate (THF) has been proposed to contribute to the assimilation of this intermediate during growth on C_1_ carbon sources such as methanol. However, the in vivo rate of this condensation reaction is unknown and its possible contribution to growth remains elusive. Here, we used microbial platforms to assess the rate of this condensation in the cellular environment. We constructed *Escherichia coli* strains lacking the enzymes that naturally produce 5,10-methylene-THF. These strains were able to grow on minimal medium only when equipped with a sarcosine (*N*-methyl-glycine) oxidation pathway that sustained a high cellular concentration of formaldehyde, which spontaneously reacts with THF to produce 5,10-methylene-THF. We used flux balance analysis to derive the rate of the spontaneous condensation from the observed growth rate. According to this, we calculated that a microorganism obtaining its entire biomass via the spontaneous condensation of formaldehyde with THF would have a doubling time of more than three weeks. Hence, this spontaneous reaction is unlikely to serve as an effective route for formaldehyde assimilation.

## 1. Introduction

Most metabolic conversions are catalyzed by dedicated enzymes or by promiscuous enzymes that evolved to catalyze other reactions [1]. Nevertheless, some reactions occur spontaneously at a sufficient rate, making enzymatic catalysis unnecessary. Spontaneous reactions seem to be prevalent in specialized metabolism, where reaction rate and specificity are of lesser importance [2]. Yet, spontaneous reactions also take place in central metabolism. For example, proline biosynthesis involves the spontaneous cyclization of glutamate 5-semialdehyde to pyrroline 5-carboxylate [3]; leucine biosynthesis involves the spontaneous decarboxylation of 2-isopropyl-3-oxosuccinate; and formaldehyde detoxification in various organisms involves the spontaneous condensation of this reactive molecule with glutathione [4].

In fact, the high reactivity of formaldehyde makes it a prime candidate for participating in spontaneous reactions. Most of these reactions are unproductive and deleterious; for example, the reaction of formaldehyde with the side-chains of arginine, cysteine, histidine, and lysine within proteins results in their inactivation and cross-linking [5]. In other cases, spontaneous reactions of formaldehyde might play a physiological role. An especially interesting case is methylotrophic microorganisms that use methanol as a sole carbon source [6]. As formaldehyde is a key intermediate in methanol assimilation, its condensation with other molecules is essential for the conversion of the C_1_ feedstock into multi-carbon metabolites. Some of the formaldehyde condensation reactions in methylotrophic organisms were speculated to be spontaneous. For example, condensation of formaldehyde with tetrahydromethanopterin, which plays a key role in the methylotrophic serine cycle [7], was once thought to proceed only spontaneously, but later an enzyme catalyzing this reaction was identified (‘formaldehyde-activating enzyme’, Fae) [8].

Another spontaneously occurring formaldehyde condensation reaction occurs with the universal C_1_ carrier, tetrahydrofolate (THF) [9]. This reaction was speculated to contribute to formaldehyde assimilation via the serine cycle [10,11,12]. However, a later study cast doubt on the physiological role of this reaction, which seems to proceed at a rate too slow to be of importance [7]. Still, although the kinetics of the spontaneous condensation of formaldehyde and THF have been measured in vitro [13], the in vivo reaction rate—that is, reaction kinetics under physiological conditions and concentrations—have never been measured. It therefore remains unclear whether this spontaneous reaction can play a physiological role in formaldehyde metabolism and assimilation.

**Figure 1:**
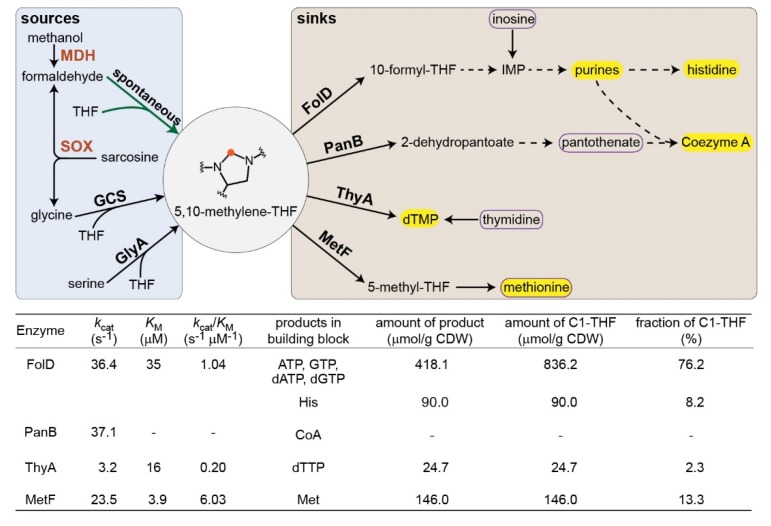
Metabolic sources and sinks for 5,10-methylene-THF. C_1_-derived biomass building blocks are shown within yellow frame, while compounds added to the medium to relieve the C_1_ auxotrophy are shown within a purple box. GCS corresponds to the glycine cleavage system. Formaldehyde is produced via the activity of either methanol dehydrogenase (MDH) or sarcosine oxidase (SOX). The kinetic parameters of the enzymes utilizing 5,10-methylene-THF [14,15,16,17] are shown together with the metabolic requirements for their products [18].

Here, we construct microbial platforms to directly assess the in vivo condensation rate of formaldehyde and THF. We generate an *Escherichia coli* strain deleted in the two enzymes that produce 5,10-methylene-THF, the key metabolic precursor of the cellular C_1_ moieties (Figure 1). Using this strain, we show that 5,10-methylene-THF can be produced in vivo via the condensation of formaldehyde and THF, but only at high formaldehyde concentrations and supporting only low growth rates. We then use computational modeling to derive the in vivo condensation rate from the measured growth rates. From the rate of this spontaneous reaction we deduce that a microbe depending solely on the condensation with THF for formaldehyde assimilation could not exceed the very low growth rate of 0.0012 h^−1^ (i.e., a doubling time of 580 h or 24 days). Hence, while the spontaneous condensation of formaldehyde and THF is feasible under in vivo conditions, its slow rate practically rules out a substantial contribution to growth on C_1_ feedstocks.

## 2. Results

**Figure 2:**
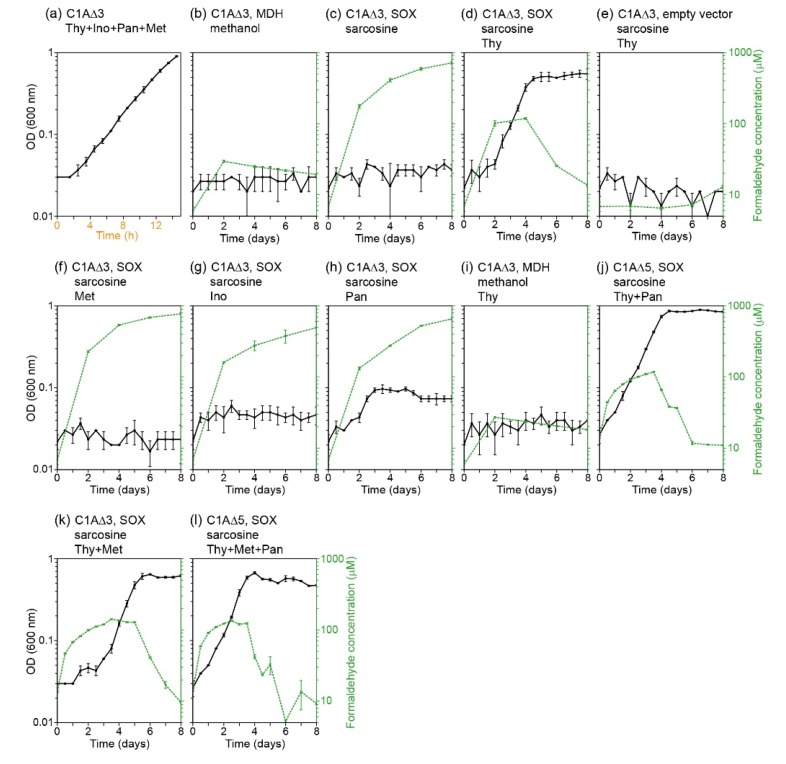
Growth of the C1AΔ3 and C1AΔ5 strains with formaldehyde as source of 5,10-methylene-THF. The strain and carbon sources used in each experiment are shown above each box. Note that the scale of x-axis in panel (a) is different from the others. C1AΔ3 corresponds to Δ*glyA* Δ*gcvTHP* Δ*frmRAB*, C1AΔ5 corresponds to Δ*glyA* Δ*gcvTHP* Δ*frmRAB ΔpanB* Δ*ltaE*, MDH to methanol dehydrogenase, and SOX to sarcosine oxidase (Table 1). Strains were cultivated on a minimal medium with 10 mM glucose, supplemented either with 1 mM sarcosine or with 700 mM methanol + 1 mM glycine, and further supplemented (as indicated in the title) with 0.3 mM thymidine (Thy), 0.3 mM methionine (Met), 0.3 mM inosine (Ino), or 6 μM pantothenate (Pan). Solid black lines correspond to OD_600_, while dashed green lines indicate formaldehyde concentrations. Error bars represent standard deviations with *n* = 3.

In *E. coli*, as in many other organisms, the essential cellular C_1_ moieties are derived from 5,10-methylene-THF (Figure 1). This metabolite is produced either via serine cleavage, as catalyzed by serine hydroxymethyltransferase (encoded by *glyA* in *E. coli*), or via glycine cleavage, as catalyzed by the glycine cleavage system (GCS, encoded by *gcvTHP* in *E. coli*) [19]. We constructed a Δ*glyA* Δ*gcvTHP* strain, auxotrophic for glycine and all cellular C_1_ moieties [20]. This strain could grow on a minimal medium only if provided with glycine and several compounds whose biosynthesis requires a C_1_ moiety (Figure 2a, growth rate of 0.25 h^−1^ ± 0.007, SD with *n* = 3): inosine, as a source of purines and histidine; pantothenate, as a source of coenzyme A; thymidine; and methionine (boxed in purple in Figure 1).

We reasoned that the in vivo condensation with THF would enable formaldehyde to serve as a precursor for 5,10-methylene-THF, making the addition of the C_1_-derived compounds redundant (Figure 1). As direct addition of formaldehyde to the medium is restricted to very low concentrations, due to its toxicity, we decided to explore the addition of compounds that can be converted to formaldehyde in situ [21]. We explored both methanol and sarcosine (i.e., *N*-methyl-glycine) as precursors of formaldehyde. Methanol can be oxidized to formaldehyde via the activity of methanol dehydrogenase (MDH), although the reaction rate is highly constrained by unfavorable thermodynamics and kinetics [21]. On the other hand, sarcosine can be converted irreversibly to formaldehyde (and glycine) via the activity of sarcosine oxidase (SOX), an enzyme characterized by a high rate [22]. Indeed, in a previous study, we found that while the methanol-dependent system enabled in vivo formaldehyde accumulation up to 50 μM only, the sarcosine-dependent system supported formaldehyde accumulation to concentrations higher than 500 μM [21]. Such formaldehyde accumulation relies on the disruption of the glutathione-dependent formaldehyde-oxidation pathway (Δ*frmRAB*), which otherwise depletes the pool of this reactive intermediate [21].

We therefore constructed a Δ*glyA* Δ*gcvTHP* Δ*frmRAB* strain, which we termed C1AΔ3 (C_1_ auxotroph with three gene/operon deletions), in which we expressed either MDH or SOX (Table 1). Neither enzyme was able to support growth without the added C_1_-derived compounds when supplemented with glycine and methanol or sarcosine, respectively (Figure 2b,c). However, the C1AΔ3 strain expressing SOX was able to grow when supplemented with sarcosine and thymidine (Figure 2d). This indicates that the in vivo condensation of formaldehyde and THF can generate 5,10-methylene-THF for the biosynthesis of all C_1_-derived compounds except thymidine. Growth was not possible when SOX was not expressed (Figure 2e), confirming that thymidine itself cannot provide 5,10-methylene-THF for growth.

**Figure 3:**
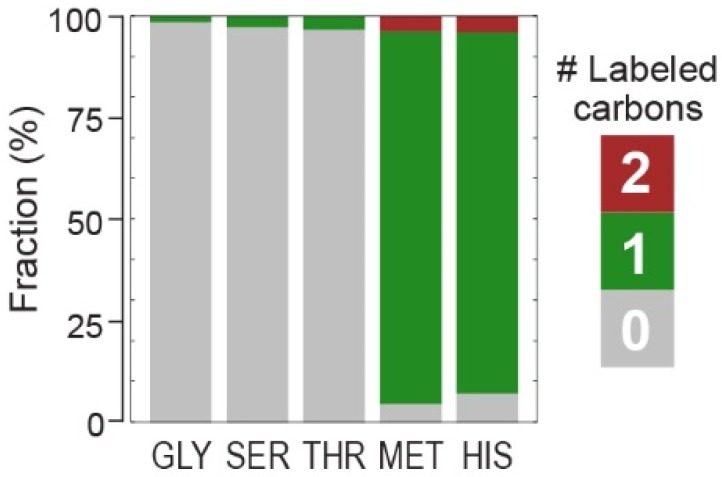
^13^C-labeling confirms that 5,10-methylene-THF is produced solely from formaldehyde. Labeling pattern of proteinogenic glycine (GLY), serine (SER), threonine (THR), methionine (MET), and histidine (HIS) upon feeding the C1AΔ3 strain with unlabeled glucose, glycine, thymidine, as well as sarcosine-(methyl-^13^C). Methionine and histidine contain a carbon derived from 5,10-methylene-THF and thus are fully singly labeled, while glycine, serine, and threonine serve as a control.

To unequivocally confirm that 5,10-methylene-THF is derived from formaldehyde, we conducted a ^13^C-labeling experiment. We fed the strain with thymidine and sarcosine-(methyl-^13^C) and measured the labeling profile of proteinogenic amino acids. As expected, we found methionine and histidine, both harboring a carbon that is derived form 5,10-methylene-THF [20], to be singly labeled. Glycine, serine, and threonine, serving as a control, were unlabeled (Figure 3).

Unlike with thymidine, the addition of methionine, inosine, or pantothenate separately did not support growth even in the presence of sarcosine (Figure 2f–h). Why is the addition of thymidine necessary for growth while the other C_1_-derived compounds can be produced from formaldehyde? As thymidine biosynthesis requires a considerably lower amount of 5,10-methylene-THF than that required for the biosynthesis of purines, histidine, and methionine (Figure 1), the reason cannot be related to an especially higher metabolic burden of diverting 5,10-methylene-THF towards the production of thymidine. Instead, the kinetics of the different enzymes that use 5,10-methylene-THF may explain the requirement for thymidine supplementation. Among these enzymes, thymidylate synthase (ThyA) has the lowest reported *k_cat_* and *k_cat_*/*K*_M_ values with respect to 5,10-methylene-THF (Figure 1). Hence, it is probably outcompeted by the other enzymes for the use of 5,10-methylene-THF produced from formaldehyde, rendering the cell auxotrophic for thymidine and thus non-viable [25].

The concentration of formaldehyde differed substantially according to the conditions. In conditions which did not support growth, the continuous oxidation of sarcosine by the cells present in the inoculation resulted in a monotonic increase in formaldehyde concentration, reaching 1000 μM (Figure 2c,f–h). However, in the growing culture, formaldehyde concentration plateaued at 100 μM during exponential growth, and then decreased during the stationary phase (Figure 2d). This indicates active metabolism of formaldehyde by the cells.

Notably, the strain expressing MDH was not able to grow when supplemented with glycine, methanol and thymidine (Figure 2i). As a formaldehyde concentration of ≥ 100 μM seems to be necessary for a sufficiently high rate of its spontaneous condensation with THF (Figure 2d), the inability to grow with methanol could be explained by the low formaldehyde concentration observed with methanol oxidation, 20–30 μM (Figure 2b,i).

It is possible that the condensation of formaldehyde and THF is catalyzed promiscuously by an *E. coli* enzyme, thus masking the spontaneous reaction. Reviewing *E. coli* native enzymes, we identified two that might be able to catalyze the condensation reaction promiscuously: (i) 3-methyl-2-oxobutanoate hydroxymethyltransferase (PanB), some variants of which are known to condense formaldehyde and 3-methyl-2-oxobutanoate to give 2-dehydropantoate [26], which could then donate a C_1_ moiety to THF via the reversal of the major enzyme activity. (ii) Threonine aldolase (LtaE), an enzyme evolutionarily related to serine hydroxymethyltransferase, which might be able to promiscuously accept formaldehyde and THF [27].

We therefore constructed a Δ*glyA* Δ*gcvTHP* Δ*frmRAB* Δ*panB* Δ*ltaE* strain, which we termed C1AΔ5 (C_1_ auxotroph with five gene/operon deletions, Table 1). Due to the deletion of *panB*, this strain requires the addition of pantothenate to the medium regardless of the presence of formaldehyde. The C1AΔ5 strain expressing SOX displayed the same growth phenotype as the C1AΔ3 strain when cultivated on sarcosine and thymidine (and pantothenate in the case of the former strain, Figure 2j). This indicates that the observed growth is unlikely to be due to promiscuous enzyme catalysis but rather corresponds to spontaneous condensation of formaldehyde and THF (although we cannot completely rule out the existence of an uncharacterized enzyme catalyzing this reaction).

Since the growth rate observed with formaldehyde serving as a precursor of 5,10-methylene-THF and downstream metabolites (Figure 2d,j) was substantially lower than that observed when all C_1_-derived compounds were added to the medium (Figure 2a), it is clear that the spontaneous condensation of formaldehyde and THF limits growth. Therefore, there should exist a direct relationship between the formaldehyde condensation flux and the growth rate, such that the former can be deduced from the latter. Moreover, assuming a fixed rate of formaldehyde condensation with THF, the growth rate should be proportional to the metabolic requirement for 5,10-methylene-THF (further assuming that C_1_-THF is not “wasted”, e.g., via the activity of 10-formyl-THF deformylase). Importantly, in a previous study, we found that the addition of ≤5 mM sarcosine to a Δ*frmRAB* strain expressing SOX did not impair growth [21], thus confirming that the accumulated formaldehyde in this case is not toxic and does not influence the observed growth rate.

The growth rate observed without the addition of pantothenate, 0.037 h^−1^ ± 0.007 (SD with *n* = 9), was not significantly different from that observed with pantothenate, 0.035 h^−1^ ± 0.005 (SD with *n* = 9; *p*-value > 0.4, *t*-test). This is expected from the minor contribution of coenzyme A to biomass, such that the metabolic requirement for 5,10-methylene-THF changes only negligibly with the addition of pantothenate. Overall, the growth rate observed with thymidine, with or without pantothenate, was 0.036 h^−1^ ± 0.006 (SD with *n* = 18).

**Figure 4:**
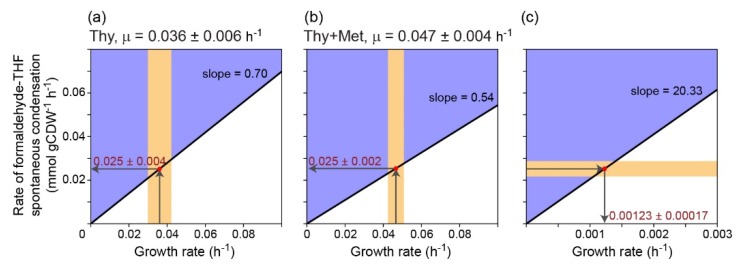
Computational modeling of the relationship between the growth rate and the rate of the spontaneous condensation of formaldehyde and THF. Shown are the phenotypic phase planes of the formaldehyde condensation reaction, when (**a**) thymidine (Thy) is added to the medium; or when (**b**) thymidine and methionine (Thy + Met) are added to the medium. (**c**) Phenotypic phase plane of the formaldehyde condensation reaction when this reaction provides the sole source of carbon via the serine cycle.

To derive the formaldehyde condensation rate from this growth rate we used a metabolic model of *E. coli* [28], from which we deleted the reactions that natively produce 5,10-methylene-THF and added a reaction that condenses formaldehyde with THF (Methods). We assumed glucose, glycine, formaldehyde, and thymidine as carbon sources and used Flux Balance Analysis (FBA) to calculate the phenotypic phase plane [29] of the formaldehyde condensation reaction. The slope of the lower bound of the phenotypic phase plane represents the expected relationship between the growth rate and the formaldehyde condensation rate: 0.7 mmol gCDW^−1^ (Figure 4a; adding pantothenate barely changed the phenotypic phase plane, due to the negligible contribution of coenzyme A to biomass). By intersecting the phenotypic phase plane with the measured growth rate, we derived a formaldehyde condensation rate of 0.025 mmol gCDW^−1^ h^−1^ ± 0.004 (Figure 4a). We note that this value corresponds to the net reaction rate, that is, the rate of the condensation reaction minus the dissociation rate of 5,10-methylene-THF.

To check the robustness of this derivation, we decided to repeat it, cultivating the SOX expressing strains, both C1AΔ3 and C1AΔ5, in the same conditions as before, while further adding methionine to the medium (Figure 2k, Figure 2l, respectively). The addition of methionine is expected to substantially decrease the metabolic requirement for 5,10-methylene-THF, thus supporting a higher growth rate. Indeed, the growth rate under these conditions, 0.047 h^−1^ ± 0.004 (SD with *n* = 18) was significantly higher than that observed without the addition of methionine (*p*-value < 10^−5^, *t*-test; as before, addition or omission of pantothenate did not significantly change the growth rate, *p*-value > 0.1, *t*-test).

We used FBA again to calculate the phenotypic phase plane of the formaldehyde condensation reaction when methionine is also added to the medium. We found that the correlation factor between the growth rate and the formaldehyde condensation rate (i.e., the slope of the phenotypic phase plane, Figure 4b) decreased to 0.54 mmol gCDW^−1^, as expected from the lower metabolic requirement for 5,10-methylene-THF when methionine is added to the medium. By intersecting the new phenotypic phase plane with the growth rate measured with methionine addition, we derived a formaldehyde condensation flux of 0.025 mmol gCDW^−1^ h^−1^ ± 0.002 (Figure 4b), identical to what was previously calculated.

The fact that we derived an identical formaldehyde condensation rate using different growth conditions that are characterized by different growth rates validates our approach and assumptions, especially confirming that the condensation rate does not change between conditions and that the growth rate is directly related to the metabolic requirement for 5,10-methylene-THF.

**Figure 5:**
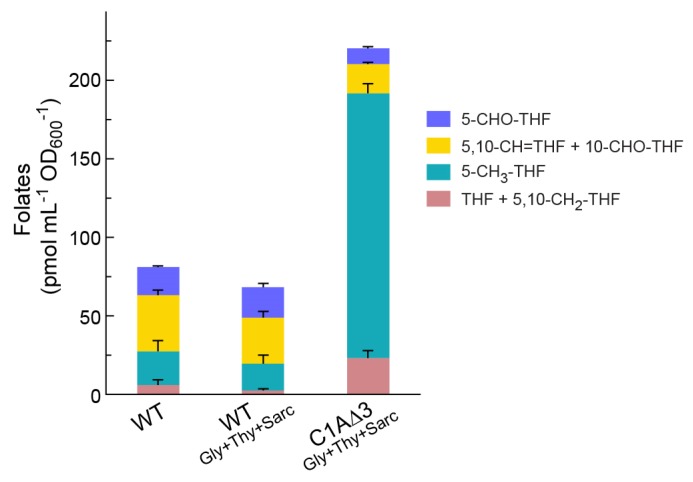
Intracellular content of THF and its C_1_ derivatives. Strains were cultivated on a minimal medium with 10 mM glucose, supplemented with 1 mM glycine (Gly), 0.3 mM thymidine (Thy), and 1 mM sarcosine when indicated. Error bars represent standard errors, with *n* = 3.

To better understand the metabolic adaptation to the novel 5,10-methylene-THF biosynthesis route, we quantified the intracellular concentrations of THF and its C_1_ derivatives by UPLC (Methods). We found the total THF pool in the C1AΔ3 strain to be almost 3-fold higher than in a WT strain (Figure 5). This might reflect a feedback mechanism, in which the low rate at which C_1_-THFs are produced led the cell to increase the biosynthesis of the cofactor. Interestingly, the fraction of unbound THF does not seem to be higher in the C1AΔ3 strain, as [THF] + [5,10-methylene-THF] (which our analysis cannot separate) represents ≤ 10% of the total THF pool both in the WT strain and in the C1AΔ3 strain. However, the THF pool in the C1AΔ3 strain is strongly shifted towards a more reduced state (Figure 5). While the oxidized 5-formyl-THF, 10-formyl-THF, and 5,10-methenyl-THF constitute 66% of the total THF pool in the WT strain, they represent only 13% of the total in the C1AΔ3 strain. In contrast, the reduced 5-methyl-THF constitutes only 27% of the THF pool in the WT strain, but represents 77% of the THF pool in the C1AΔ3 strain.

How does the in vivo derived condensation flux compare with that expected using the in vitro measured parameters? Using the previously measured kinetic parameters, the rate of 5,10-methylene-THF formation equals *k*_forward_ × [formaldehyde] × [THF] × [H^+^], where *k*_forward_ = 8.7 × 10^8^ s^−1^ M^−2^ [13]. Even if we assume that the THF/5,10-methylene-THF pool (Figure 5) exists only as free THF, such that [THF] = 23 pmol mL^−1^ OD_600_^−1^ = 18 µM (considering culture biomass of 0.39 mgCDW mL^−1^ OD_600_^−1^ and intracellular biomass of 300 mgCDW mL^−1^ [30]), further assuming [formaldehyde] = 100 µM (Figure 2d,j–l) and [H^+^] ≈ 32 nM (pH 7.5), we get a 5,10-methylene-THF formation rate of only 0.00058 mmol gCDW^−1^ h^−1^, that is, 43-fold lower than the in vivo derived rate. Since [THF] should be lower than [THF] + [5,10-methylene-THF], and the reverse reaction (i.e., 5,10-methylene-THF cleavage) further lowers the net formaldehyde condensation flux, the in vivo condensation flux seems likely to be 2–3 orders of magnitude higher than that expected from the in vitro parameters. While the intracellular concentration of formaldehyde might be somewhat higher than that measured in the medium, it is highly unlikely that this discrepancy accounts for the 2–3 orders of magnitude difference. Hence, it is rather clear that under cellular conditions, the condensation rate of formaldehyde with THF is substantially higher than that measured in simple model in vitro systems, further emphasizing the importance of the analysis performed here.

Next, we asked what would be the growth rate of an organism growing on formaldehyde (or a precursor thereof) using the serine cycle and assuming that 5,10-methylene-THF is produced solely via the spontaneous condensation of formaldehyde with THF. To assess this rate, we added to the model of *E. coli* the formaldehyde-THF condensation reaction as well as the specific reactions of the serine cycle (e.g., malyl-CoA synthetase and lyase, serine transaminase, see Methods). Again, we calculated the phenotypic phase plane of the formaldehyde condensation reaction, assuming formaldehyde to be the sole carbon source. As expected, the correlation factor between the growth rate and the formaldehyde condensation rate was much higher than before, >20 mmol gCDW^−1^ (Figure 4c, note the different scaling of the x-axis). By intersecting the phenotypic phase plane with the formaldehyde condensation rate calculated above, we derived a maximal growth rate of 0.0012 h^−1^ ± 0.0002. (In fact, this growth rate can also be estimated directly from the growth rate of the C1AΔ3 strain, which uses formaldehyde as a source of the non-thymidine C_1_ moieties (Figure 2d,j, 0.036 h^−1^): as 2.3% of the carbons in biomass are derived from non-thymidine C_1_ moieties [18], the expected growth rate when the condensation reaction provides 100% of the carbons is expected to be 0.036 × (2.3%/100%) = 0.0008 h^−1^, very close to the value derived by the computational modeling.) This growth rate is equivalent to a doubling time of 580 h, i.e., 24 days. Obviously, such a growth rate is impractical for most microorganisms, ruling out the spontaneous condensation of formaldehyde and THF as an effective route for formaldehyde assimilation.

## 3. Discussion

In this study, we constructed microbial platforms to assess the in vivo rate of the spontaneous condensation of formaldehyde with THF. We were able to select for this reaction as the sole source of 5,10-methylene-THF and downstream metabolites, but only when (i) sarcosine oxidation sustained a high concentration of formaldehyde and (ii) thymidine was added to the medium. The failure to obtain growth with methanol as a source of formaldehyde might be attributed to the use of an NAD-dependent MDH, which strongly favors formaldehyde reduction over methanol oxidation. A quinone-dependent MDH or an O_2_-dependent methanol oxidase, both strongly favoring methanol oxidation, is expected to enable growth with methanol just as SOX enabled growth with sarcosine. The strict necessity for thymidine supplementation could be attributed to the poor kinetics of the enzyme that generates it from 5,10-methylene-THF. This kinetic constraint does not seem to hamper thymidine biosynthesis in wild-type *E. coli* presumably because the biosynthesis of 5,10-methylene-THF is not limiting. In our strain, the low production rate of 5,10-methylene-THF likely generates a “competition” between its various metabolic sinks (Figure 1), such that the kinetically poor ThyA cannot sustain sufficiently high flux to support growth.

As we demonstrated, a microorganism obtaining its entire biomass via the spontaneous condensation of formaldehyde with THF would be constrained to a very low growth rate, having a doubling time of more than three weeks. However, it is important to remember that the spontaneous condensation rate might differ between organisms. For example, it could be improved by increasing the cellular concentration of THF or operating at a higher formaldehyde concentration. Moreover, changing the cellular conditions—temperature, pH, ionic strength, and concentrations of different ions—might alter the rate at which formaldehyde reacts with THF. Whether such changes could substantially increase the spontaneous production rate of 5,10-methylene-THF, thus enabling a more reasonable growth rate, remains to be seen. It will be interesting to use adaptive laboratory evolution [31] to try to increase the spontaneous condensation rate. For example, the *E. coli* strains established here could be cultivated in a turbidostat regime [32] selecting for changes in cellular conditions that support a higher 5,10-methylene-THF production rate, thus increasing the growth rate. Alternatively, it is at least possible that a variant of the formaldehyde activating enzyme [8] has evolved, or could be evolved, to accept THF rather than tetrahydromethanopterin, thus enabling a dramatic increase in the rate of 5,10-methylene-THF production from formaldehyde.

**Figure 6:**
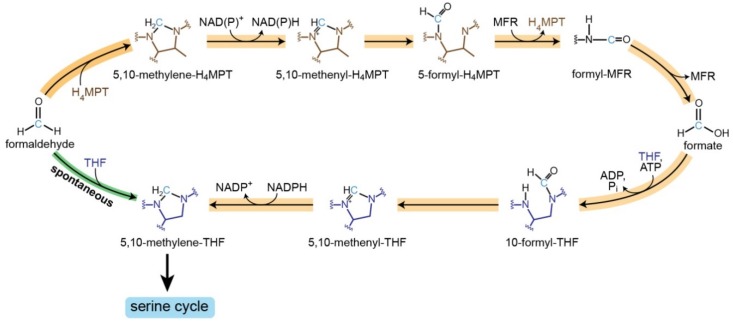
Spontaneous formaldehyde THF condensation bypasses the long and ATP costly tetrahydromethanopterin (H_4_MPT)-dependent route.

Despite the kinetic barrier, assimilation of formaldehyde via its spontaneous condensation with THF might be advantageous in conditions that favor high biomass yield rather than growth rate. This is due to two reasons. First, microorganisms that grow on methanol via the serine cycle use a long, tetrahydromethanopterin-dependent route (Figure 6). Bypassing this route via the direct condensation of formaldehyde and THF saves the need for the costly biosynthesis of multiple proteins as well the additional cofactor. Moreover, the direct condensation route saves the ATP consumed to energize the condensation of formate with THF (Figure 6). Hence, while unlikely, we cannot rule out the possibility of a slowly growing methylotrophic microorganism, living in a resource-deprived environment, which operates the serine cycle via the spontaneous formaldehyde-THF condensation in order to save cellular resources and maximize biomass yield. We leave it to future studies to explore the possible existence of such an interesting growth mode.

## 4. Materials and Methods

### 4.1. Strains and Genomic Modifications

All strains used in this study are listed in Table 1. An *E. coli* K-12 MG1655 derived strain SIJ488 [23] was used as the parental strain for genomic modifications and further experiments. Multiple-gene knockouts were obtained by iterative rounds of P1 phage transduction [33] and antibiotic resistant gene cassette flip-out. Donor lysates of the transduction were prepared using “_don” strains. Gene knockouts were confirmed by PCR after antibiotic selection. Antibiotic cassette was removed by inducing Flippase [23]. Colonies growing only on plates without antibiotic were further confirmed by PCR of the cassette removal. Codon optimized methanol dehydrogenase (MDH) from *Corynebacterium glutamicum* R and sarcosine oxidase (SOX) from *Bacillus sp.* B-0618 were overexpressed in a pZASS plasmid, which has a p15A origin, a constitutive promoter pgi-20, and a streptomycin selection marker; gene sequences and cloning details were provided previously in ref. [21].

### 4.2. Media and Growth Conditions

LB medium (1% NaCl, 0.5% yeast extract, and 1% tryptone) was used for genomic strain modifications. Antibiotics were used at the following concentrations: kanamycin, 50 μg/mL and streptomycin, 100 μg/mL. Growth experiments were performed in M9 minimal media (47.8 mM Na_2_HPO_4_, 22 mM KH_2_PO_4_, 8.6 mM NaCl, 18.7 mM NH_4_Cl, 2 mM MgSO_4_, and 100 μM CaCl_2_), supplemented with trace elements (134 μM EDTA, 31 μM FeCl_3_·6H_2_O, 6.2 μM ZnCl_2_, 0.76 μM CuCl_2_·2H_2_O, 0.42 μM CoCl_2_·2H_2_O, 1.62 μM H_3_BO_3_, and 0.081 μM MnCl_2_·4H_2_O). Additional 50 μM MnCl_2_ was added to relieve possible oxidative stress from sarcosine oxidation [34]. 10 mM glucose was the main carbon source and 1 mM of glycine was used to relieve the glycine auxotroph. 1 mM sarcosine or 700 mM of methanol was used as the formaldehyde source, C_1_ supplements were added according to the specific experiment: 0.3 mM of thymidine, 0.3 mM of inosine, 0.3 mM of methionine, and 6 µM of pantothenate [20]. We note that the very high concentration of methanol was required for formaldehyde production because methanol oxidation is thermodynamically unfavorable and must be pushed forward. On the other hand, sarcosine oxidation is highly favorable, such that even 1 mM of this precursor is sufficient to sustain the efficient production of formaldehyde.

Strains were precultured in 4 mL M9 minimal medium with all C_1_ supplements, as well as glucose and glycine. The precultures were harvested and washed three times in M9 medium, then inoculated into 4 mL M9 media with the different carbon sources as explained for each experiment at a starting OD_600_ of 0.02. The same preculture was used to inoculate the three experimental replicates. The cultures were incubated at 37 °C, 200 rpm shaken (GFL Orbital Shaker 3017, Burgwedel, Germany). Cell densities were measured by photometer (DiluPhotometer OD600, IMPLEN, Munich, Germany) at 600 nm. Cell densities at exponential phase were fitted to $\;N=N_{0}*e^{\mu \;*\;t}$ using LMFIT on python, where *N_0_* and *N* are cell densities at start and at time *t*, and *μ* is growth rate. Student’s *t-*test was used for the statistical analysis.

### 4.3. Formaldehyde Concentration Measurement

At the indicated time points, 140 μL of culture were collected and centrifuged for 10 min at 3300× *g*, 4 °C. Formaldehyde concentration in culture supernatants was determined by Nash assay [35]. 125 μL of culture supernatant was mixed with 125 μL of Nash reagent (2 M of ammonium acetate, 20 mM of acetylacetone, and 50 mM of acetic acid) in 96-well plate (Brandplates, Wertheim, Germany), and incubated at 37 °C for 1 h. The absorption at 412 nm was measured for each sample using plate reader (Infinite M200 pro, Tecan, Männedorf, Switzerland). A standard curve showed that the assay was linear at the range from 1 mM to 0 mM formaldehyde in M9.

### 4.4. Isotopic-labeling Experiments

For stationary isotope tracing of proteinogenic amino acids, C1AΔ3 strain was grown in M9 with 10 mM of glucose, 0.3 mM of thymidine, and 1 mM of either unlabeled sarcosine or methyl-^13^C labeled sarcosine (Sigma-Aldrich, Steinheim, Germany). Experiments were performed in duplicate. Cells were harvested at the late exponential phase (OD 0.5). The 2 mL of culture was harvested and washed by centrifugation. Protein biomass was hydrolyzed with 6 M HCl, at 95 °C for 24 h [36]. The samples were completely dried under a stream of N_2_ at 95 °C.

Hydrolyzed amino acids were analyzed with ultra-performance liquid chromatography coupled with electrospray mass spectrometry (UPLC–ESI–MS) as previously described [37]. Chromatography was performed with a Waters Acquity UPLC system (Waters), using an HSS T3 C_18_ reversed phase column (100 mm × 2.1 mm, 1.8 μm; Waters). Of formic acid 0.1% in H_2_O (A) and 0.1% of formic acid in acetonitrile (B) were the mobile phases. The flow rate was 0.4 mL/min and the gradient was 0–1 min—99% A; 1–5 min—linear gradient from 99% A to 82%; 5–6 min—linear gradient from 82% A to 1% A; 6–8 min—kept at 1% A; 8–8.5 min—linear gradient to 99% A; and 8.5–11 min—re-equilibrate. Mass spectra were acquired using an Exactive mass spectrometer (Thermo Scientific, Bremen, Germany) in positive ionization mode, with a scan range of 50.0–300.0 *m*/*z*. The spectra were recorded during the first 5 min of the LC gradients. Data analysis was performed using Xcalibur (Thermo Scientific, Bremen, Germany). Determination of retention times was performed by analyzing amino-acid standards (Sigma-Aldrich) under the same conditions.

### 4.5. Flux Balance Analysis

For Flux Balance Analysis and phenotypic phase plane calculations, we used the COBRApy package [38]. All scripts are available as Jupyter notebooks at https://gitlab.com/hi-he/wenzhangfujian under the directory of “2020_formaldehyde condensation”.

Adjustments made to *i*ML1515 model [28]:

- Reactions POR5 and GLYCK were removed from the model since they are not present in *E. coli* cells.
- The stoichiometry of the reaction THD2pp was changed, as the translocation uses only one proton instead of two.
- PFL and OBTFL were removed, since they are catalyzed by oxygen sensitive enzymes and therefore not active in aerobic conditions.
- Based on gathered evidence, FTHFLi was removed. This reaction is especially important in our case, since it could allow charging of THF with formate directly.
- We changed the directionality of three reactions in the nucleotide biosynthesis pathway (FTHFD, GARFT, and AICART) to be irreversible, in order to avoid nucleotides degradation to charge THF, since that is very unlikely to occur.
- We changed the thymidine periplasmic symporter reaction (THYMt3pp) to be reversible, based on our experimental evidence (adding thymidine to the media rescued growth in certain conditions).
- We knocked out the glycine cleavage system reaction and the GlyA reaction (GLYCL and GHMT2r).
- We added the formaldehyde-THF spontaneous condensation (THFSPONT) reaction: thf_c + fald_c -> mlthf_c + h2o_c.

We made further adjustments to the model to enable growth on formaldehyde via the serine cycle and ethylmalonyl-CoA cycle:

- Removed the following reactions to avoid potential carbon fixation cycles: GLXCL, GLYCL, THRD, THRA2, THRA, SERD_L.
- Added NAD-dependent formate dehydrogenase (FDH), enabling the utilization of the formate that is created from formaldehyde as source of reducing power.
- Added the crotonoyl-CoA carboxylase reaction (EtMaCoA), required for the ethylmalonyl-CoA cycle: b2coa_c + nadph_c + co2_c + q8_c + h2o_c -> glx_c + ppcoa_c + nadp_c + q8h2_c.
- Added the propionyl-CoA carboxylase reaction (MMCDr), as required for recycling the propionyl-CoA, which was created by EtMaCoA: ppcoa_c + atp_c + hco3_c -> mmcoa__S_c + adp_c + h_c + pi_c.
- Made the methylmalonyl-CoA mutase reaction (MMM) reversible so that the methylmalonyl-CoA (mmcoa__S_c) can be recycled.
- Added the reactions of the serine cycle:
  ○ serine-hydroxypyruvate amino-transferase (SGAT1): ser__L_c + akg_c -> glu__L_c + hpyr_c.
  ○ glycine-glyoxylate amino-transferase (SGAT2): gly_c + akg_c -> glx_c + glu__L_c.
  ○ malate thiokinase (MTK): mal__L_c + atp_c + coa_c -> malylcoa_c + adp_c + pi_c.
  ○ malyl-CoA lyase (MCL): malylcoa_c -> accoa_c + glx_c.

### 4.6. Folate Profiling

75 mL of each culture was incubated in 250 mL Erlenmeyer flask. At the late exponential phase, cells of each strain equivalent to approximately 5 mg total protein [30] were harvested by centrifugation (3200× *g*, 10 min, 4 °C) and resuspended in 10 mL of 50 mM HEPES-CHES (2-(N-cyclohexylamino)ethanesulfonic acid; pH 7.8 ± 0.05) containing 2% (*w*/*v*) ascorbic acid and 10 mM β-mercaptoethanol (extraction buffer). The cells were disrupted using a FastPrep Instrument (MP Biomedicals), boiled for 10 min, and centrifuged (16,000× *g*, 10 min, 4 °C). The supernatants were collected and treated with AtGGH2, as described by Ramos-Parra et al. [39]. Folates were purified by affinity chromatography and analyzed by UPLC-coupled electrochemical detector (CoulArray Model 5600A; ESA, Chelmsford, MA) as described previously [40]. Separation of folate derivatives was achieved with a Prodigy ODS(2) column (150 mm × 3.2 mm, 5 um particle size, Phenomenex, Torrance, CA, USA) using a nonlinear gradient of mobile phase A (28 mM K_2_HPO_4_ and 59 mM H_3_PO_4_) and B (75% phase A and 25% acetonitrile). Detection was achieved with an electrochemical cell adjusted to 100, 200, 300, and 400 mV, and quantification was performed using commercial standards (Schircks, Jona, Bauma, Switzerland).

## Figures and Tables

**Table 1:** List of *E. coli* strains and plasmids.

Strain	Genotype	Source
SIJ488	K-12 MG1655 Tn7::para-exo-beta-gam; prha-FLP; xylSpm-IsceI	[23]
DH5α	F^-^ *endA1 glnV44 thi-1 recA1 relA1 gyrA96 deoR nupG purB20* φ80dlacZΔM15 Δ(*lacZYA-argF*)U169, hsdR17(r_K_^-^m_K_^+^), λ^-^	Lab collection
Frm	SIJ488 *ΔfrmRAB*	[21]
gcv_don	K-12 MG1655 *ΔgcvTHP::Km*	Lab collection
ltaE_don	K-12 BW25113 *ΔltaE::Km*	[24]
panB_don	K-12 BW25113 *ΔpanB::Km*	[24]
glyA_don	K-12 BW25113 *ΔglyA::Km*	[24]
C1AΔ3	SIJ488 *ΔfrmRAB ΔgcvTHP ΔglyA::Km*	This study
C1AΔ5	SIJ488 *ΔfrmRAB ΔgcvTHP ΔltaE ΔpanB ΔglyA::Km*	This study
**Plasmid**	**Genes**	**Source**
pZASS	p15A ori; Strep^R^; P_pgi-20_	Lab collection
pZASS-soxA	pZASS::*soxA*; sarcosine oxidase	[21]
pZASS-CgadhA	pZASS::*CgadhA*; methanol dehydrogenase	[21]
